# 534. Healthcare Resource Utilization and Costs among Patients with COVID-19 in the United States

**DOI:** 10.1093/ofid/ofad500.603

**Published:** 2023-11-27

**Authors:** Gui Liu, Nicolae Done, Yan Song, Hardik Goswami, Travis Wang, Hesen Li, Matthew Mattera, James Signorovitch, Gleicy Macedo Hair

**Affiliations:** Merck & Co., Inc., Rahway, New Jersey; Analysis Group, Boston, Massachusetts; Analysis Group, Inc., Boston, Massachusetts, USA, Boston, MA; Merck & Co., Inc., Rahway, New Jersey; Analysis Group, Inc., Boston, Massachusetts; Analysis Group, Inc., Boston, Massachusetts; Analysis Group, Inc., Boston, Massachusetts; Analysis Group, Boston, Massachusetts; Merck & Co., Inc., Rahway, New Jersey

## Abstract

**Background:**

COVID-19 continued to cause significant burden on the healthcare system even during the less severe Omicron wave. We evaluated outpatient and inpatient healthcare resource use (HCRU) and costs among adults with COVID-19 in the US.

**Methods:**

Using closed claims data from HealthVerity, we identified patients aged ≥18 years with either a diagnosis or a prescription or procedure code for COVID-19 between December 24, 2021 and April 30, 2022. Index date was set as date of first diagnosis or pharmacy/procedure claim. Patients hospitalized on index date were excluded. We summarized descriptive statistics for all-cause outpatient and inpatient HCRU and costs over a 28-day follow-up, overall and by hospitalization status during follow-up, patient demographics, and selected comorbidities.

**Results:**

Of the 1,203,769 patients identified, mean age was 44 years. 48.8% of the patients had ≥2 comorbidities that increased risk for severe COVID-19, and 16.9% of the patients were immunocompromised (**Table 1**). During follow-up, 44.4% of patients had ≥1 outpatient office visit and 1.9% were hospitalized. Among patients who were hospitalized during follow-up, 18.9% were admitted to the intensive care unit (ICU) and 3.4% required invasive mechanical ventilation (IMV) (**Table 2**). Hospitalization rate was 3.8% among immunocompromised patients, with 21.8 % and 4.8% of those hospitalized requiring ICU care and IMV, respectively. Among patients with cost data, patients hospitalized during follow-up incurred a total cost of $33,329 on average, with $22,919 for inpatient services, $5,738 for outpatient services, and $4,673 in pharmacy costs. Patients not hospitalized during follow-up had mean total cost of $4,965, $2,686 for outpatient services and $2,278 for pharmacy (**Table 3**). Total cost was $13,304 for immunocompromised patients not hospitalized during follow-up and $53,036 for those hospitalized during follow-up. Total costs increased with age and number of comorbidities.

**Figure:**
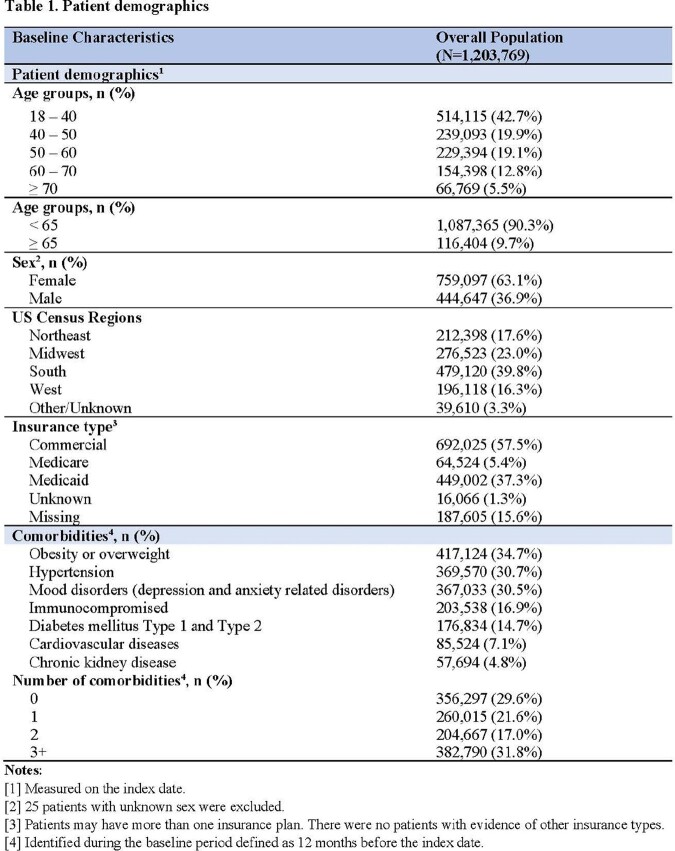

**Figure:**
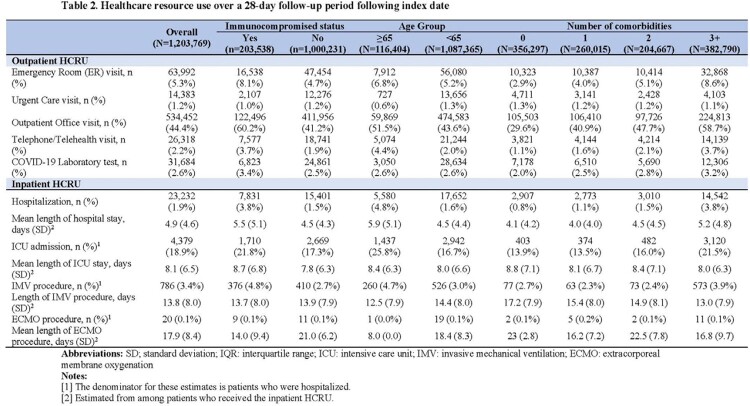

**Figure:**
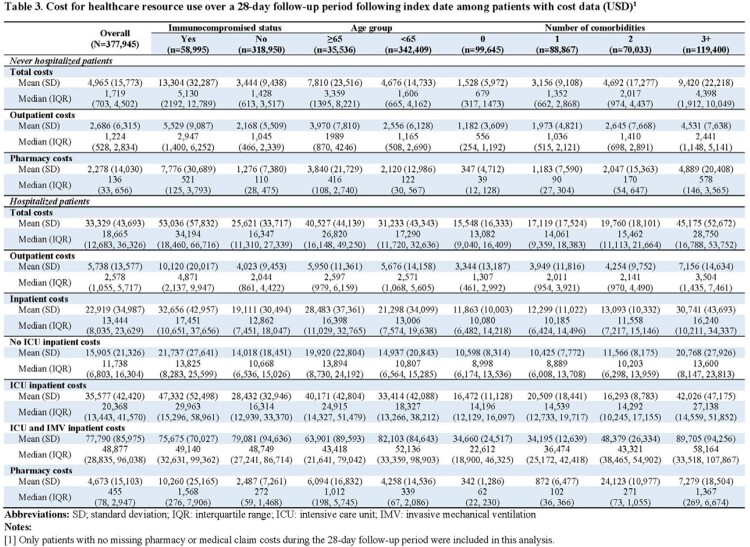

**Conclusion:**

During the Omicron wave, the cost of care for patients with COVID-19 remains high. In particular, patients with comorbidities bear substantial economic burden for their healthcare. This study provides data for future research evaluating the full economic impact of COVID-19 in the US.

**Disclosures:**

**Gui Liu, PhD**, Merck & Co., Inc.: Employee of Merck Sharp & Dohme LLC, a subsidiary of Merck & Co., Inc.|Merck & Co., Inc.: Stocks/Bonds **Nicolae Done, PhD**, Merck & Co., Inc.: Grant/Research Support **Yan Song, PhD**, Merck & Co., Inc.: Grant/Research Support **Hardik Goswami, PhD**, Merck & Co., Inc.: Employee of Merck Sharp & Dohme LLC, a subsidiary of Merck & Co., Inc.|Merck & Co., Inc.: Stocks/Bonds **Travis Wang, MS, MBBS**, Merck & Co., Inc.: Grant/Research Support **Hesen Li, PhD**, Merck & Co., Inc.: Grant/Research Support **Matthew Mattera, MPH**, Merck & Co., Inc.: Grant/Research Support **James Signorovitch, PhD**, Merck & Co., Inc.: Grant/Research Support **Gleicy Macedo Hair, PhD**, Merck & Co., Inc.: Employee of Merck Sharp & Dohme LLC, a subsidiary of Merck & Co., Inc.|Merck & Co., Inc.: Stocks/Bonds

